# Radiation-induced nanostructures: Formation processes and applications

**DOI:** 10.3762/bjnano.3.61

**Published:** 2012-07-25

**Authors:** Michael Huth

**Affiliations:** 1 Physikalisches Institut, Max-von-Laue-Str. 1, Goethe-Universität, 60438 Frankfurt am Main, Germany

**Keywords:** radiation-induced nanostructures

Radiation-induced nanostructure formation is ubiquitous. It is routinely used in lithography employing photons and masks, or in the form of focused electron beams following a maskless approach for pattern definition in a radiation-sensitive resist, also commonly known as electron beam lithography. Examples of this are found in this Thematic Series covering the topics of selected-area silicon nanowire growth by the vapor–liquid–solid approach and the preparation of monolayers of metal–organic frameworks attached to the functional groups of a self-assembled monolayer (see, e.g., [1–4]).

Not as wide-spread, but rapidly developing, is the technique of focused electron beam induced deposition (FEBID) [5]. In this technique a previously adsorbed molecular precursor is dissociated by the electron beam, leaving behind a permanent deposit of an amorphous, nanogranular [6–7] or polycrystalline microstructure with a minimum feature size well below 10 nm. Selected aspects of this technique and its application are reviewed in this Thematic Series. In a somewhat analogous fashion, swift heavy ions can be used as nanopore-forming, seeding probes. When passing through thin polymer foils they leave behind a damage track, which can be further processed to form nanopores or nanochannels to be applied in biochemical analytics or as templates for the (galvanic) growth of metallic or semiconducting nanowires, as is also reviewed in one of the following contributions. In close connection to this, highly energetic particles, in particular those from the sun, pose a risk for astronauts as they can induce severe DNA damage upon passing through body tissues. On the other hand, this same observation has led to the rise of charged-particle cancer therapy over the past 20 years.

Conceptually speaking, electrons that locally drive molecular dissociations, as well as swift heavy ions that locally cause damage in polymers or living tissue, define a principle of nanostructure formation by destructive means. But there is a deeper connection on the microscopic level.

In FEBID the dominating contribution to the dissociation yield stems from low-energy electrons in the energy range between a few to several hundred electron volts. Different processes, such as dissociative electron attachment, neutral dissociation or dissociative ionization act together in breaking selected bonds in (mostly) metal–organic precursor molecules. On the other hand, low-energy electrons also play a role in the radiation damage induced by ionizing radiation in living tissue, which causes different types of DNA damage on bases, as well as single- and double-bond breaks. High-energy particles travel in straight trajectories and have a relatively well-defined stopping point, at which the majority of the energy is deposited. In the tracks, the linear energy transfer, i.e., the rate at which ionization is created along the particle trajectory, can amount to more than 100 keV/µm. Proximal tissue, in contrast, only receives radiation by means of the excited secondary electrons whose trajectories are transverse to the particle track. For the electrons in the low-energy part of the energy spectrum, i.e., below about 5 keV, the biological effectiveness increases strongly. This increased effectiveness indicates a parallel to the FEBID process and points towards an analogous increase in the dissociation cross section at low electron energies.

A full microscopic understanding of the different dissociation pathways and bond-breaking mechanisms would be highly valuable. On the one hand, for FEBID this holds the promise of developing this technique towards electron-controlled chemistry on the nanometer scale. For cancer therapy and the understanding of DNA damage, a deeper insight into the biological effectiveness and long-term risks caused by low-energy electrons could be expected. On the theoretical level, this poses a highly complex problem on multiple scales, ranging from the sub-nanometer to the mesoscopic range, at time scales from femtoseconds to microseconds. This can only be mastered with a broad basis of different experimental and theoretical methods. For the latter, this comprises the development of new theoretical approaches that show reliable scaling behavior, and the application of established state-of-the-art methods for a proper description of the relevant vibronic and electronic degrees of freedom when molecules are organized to form larger complexes.

Research in the fields of FEBID, electron-controlled chemical lithography, radiation biophysics, and nanowires or nanochannels is conducted in a range of different communities. This Thematic Series is intended to provide a forum that brings together selected contributions from these fields. It is hoped that the reader originally interested in only one of the presented topics may be willing to digress a bit from his or her usual main path and take a look into the other research areas. Ultimately, the connections between these fields, as alluded to above, may receive some appreciation and will eventually lead to a mutual reinforcement and fruitful developments.

Michael Huth

Frankfurt, July 2012
